# Middle Cerebellar Peduncle Width—A Novel MRI Biomarker for FXTAS?

**DOI:** 10.3389/fnins.2018.00379

**Published:** 2018-06-25

**Authors:** Annie L. Shelton, Jun Y. Wang, Emily Fourie, Flora Tassone, Anna Chen, Lauren Frizzi, Randi J. Hagerman, Emilio Ferrer, David Hessl, Susan M. Rivera

**Affiliations:** ^1^MIND Institute, University of California Davis Medical Center, Sacramento, CA, United States; ^2^Center for Mind and Brain, University of California, Davis, Davis, CA, United States; ^3^Department of Psychology, University of California, Davis, Davis, CA, United States; ^4^Department of Biochemistry and Molecular Medicine, University of California, Davis, Sacramento, CA, United States; ^5^Department of Pediatrics, University of California Davis Medical Center, Sacramento, CA, United States; ^6^Department of Psychiatry and Behavioral Sciences, University of California Davis Medical Center, Sacramento, CA, United States

**Keywords:** fragile X, FXTAS, biomarkers, MRI, middle cerebellar peduncle

## Abstract

Fragile X-associated tremor/ataxia syndrome (FXTAS) is a severe neurodegenerative movement disorder affecting over 40% of male and 16% of female *FMR1* premutation carriers over the age of 50. However, there is a lack of prognostic biomarkers to aid early diagnosis and treatment planning. Therefore, this study aimed to assess the utility of the Magnetic Resonance Parkinson Index (MRPI) as a potential MRI biomarker for FXTAS. The four measurements required for the MRPI were assessed in 45 male premutation carriers at risk of developing FXTAS (Mean age = 59.54 years), 53 male patients with FXTAS (Mean age = 66.16 years) and 61 male controls (Mean age = 60.75 years), of which 73 participants had follow-up visits on average 1.96 years later. Middle cerebellar peduncle (MCP) width as well as midbrain and pons cross-sectional area were reduced in patients with FXTAS compared to both premutation carriers without FXTAS and controls. While these measurements were not found to change over time in the three-group analysis, age was an important predictor of midbrain cross-sectional area and pons/midbrain ratio. MCP width was initially reduced in a subset of premutation carriers who developed FXTAS symptoms between their initial and follow-up visits, which also decreased between visits, compared to age-matched premutation carriers who did not show any FXTAS symptom development over time. Therefore, while the MPRI may not be a useful biomarker for FXTAS, decreased MCP width may be one of the first notable signs of FXTAS, and therefore the first biomarker with the potential to identify those most at risk for the disorder.

## Introduction

Fragile X-associated tremor/ataxia syndrome (FXTAS) is a severe neurodegenerative movement disorder caused by premutation expansions (55–200 CGG repeats) in the 5′ untranslated region of the fragile X mental retardation 1 (*FMR1*) gene, located on the X-chromosome. An estimated 1 in 209 females and 1 in 430 males carry a premutation allele (Tassone et al., 2012). Studies suggest that ~40% of male and 8–16% of female premutation carriers over the age of 50 years (Rodriguez-Revenga et al., 2009) have FXTAS, but these figures rise to 75% of male premutation carriers over the age of 70 years (Jacquemont et al., 2004). Despite the relatively high incidence, there is currently a lack of prognostic markers to detect the earliest neurodegenerative signs of FXTAS.

The diagnostic criteria of FXTAS comprise three domains: clinical, radiological, and pathological (Jacquemont et al., 2003; Hall et al., 2014). Whether definite, probable or possible, FXTAS diagnosis requires a degree of functional/clinical impairment. While the clinical presentation is variable, the initial presenting motor symptom is often an intention or kinetic tremor, followed by cerebellar ataxia, both of which typically progress over time (Apartis et al., 2012). Other clinical features may include executive dysfunction, ranging from mild to dementia-like in severity, which often presents later in the disease course (Seritan et al., 2008).

The neuropathology of FXTAS is characterized by the presence of ubiquitin-positive intracellular inclusions in neural and astrocyte cells within the cortex and cerebellum in post-mortem analysis (Greco et al., 2002). Currently, FXTAS neuropathology can only be confirmed after death and hence cannot be used as a prognostic tool.

The radiological phenotype emphasizes FXTAS as a white matter neurodegenerative disease, as prior studies have highlighted the presence of white matter lesions in the middle cerebellar peduncles (MCP), known as the MCP sign, and brainstem, as well as generalized cerebral lesions and atrophy throughout the neocortex (Cohen et al., 2006; Rivera et al., 2010; Hashimoto et al., 2011). Indeed, it has been suggested that hyperintensities of the splenium of the corpus callosum should be added as a minor criterion for FXTAS, given that these lesions were found to be more common than MCP lesions in female premutation carriers with FXTAS (Apartis et al., 2012). The MCP sign is not specific to FXTAS (Storey and Billimoria, 2005) and is occasionally seen in other rare neurodegenerative diseases such as sporadic olivopontocerebellar atrophy and spinocerebellar ataxia (Brunberg et al., 2002; Quattrone et al., 2016).

Hence, the radiological criteria for FXTAS are continually evolving, and we are still lacking a single marker that can sensitively and specifically predict FXTAS onset or severity. The Magnetic Resonance Parkinson Index (MRPI) has been shown to successfully distinguish subcortical movement disorders, such as Parkinson's disease and progressive supranuclear palsy, both of which present with a tremor similar to that seen in FXTAS (Quattrone et al., 2008). Not only has the MRPI been shown to differentiate these disorders, but it has been found to be predictive of vertical supranuclear gaze palsy in patients affected by progressive supranuclear palsy over time (Okamoto et al., 2003). The MRPI is a ratio that encompasses measurements of the MCP and superior cerebellar peduncle (SCP) widths, as well as the cross-sectional area of the midbrain and pons; MRPI = [(Pons area/Midbrain area) x (MCP width/SCP width)]. These regions play an integral role within the cortico-cerebellar pathway, which is necessary for the learning and coordination of various movements (Ramnani, 2006). Therefore, these measurements and their ratios may prove to be useful biomarkers for FXTAS, a neurodegenerative movement disorder, given that MCP hyperintensities are a radiological sign of FXTAS, and there are anecdotal reports of the hummingbird sign (the ratio of the cross-sectional area of the midbrain and pons) being present in some patients with FXTAS. Therefore, the aim of this study was to assess the utility of these MRPI measurements and ratios as radiological biomarkers for FXTAS, both in early and later stages of the disorder.

## Materials and methods

### Participants

A total of 159 male participants over the age of 40 years were recruited from the Sacramento, CA area and throughout the United States and Canada as part of two continuing and concurrent longitudinal studies (PIs: Hessl and Rivera; Hagerman). The Institutional Review Board of UC Davis approved this protocol and all participants gave written informed consent before participating in the study in line with the Declaration of Helsinki. CGG repeat length and neurological assessment resulted in three experimental groups of participants at visit 1: healthy controls (*n* = 61), premutation carriers without FXTAS (*n* = 45; FXTAS stage score of 0 to 1—no or equivocal tremor/ataxia), and premutation carriers with FXTAS (*n* = 53; FXTAS stage scale score ≥2—clear tremor/ataxia with some interference in functioning). FXTAS stage scores were based on the clinical descriptions previously defined (Bacalman et al., 2006). All participants were scanned at visit 1 and 73 had a follow-up scan on average 1.96 years later (standard deviation = 0.88, range 0.75–4.79 years) (Table 1 for demographics). Twenty-five were premutation carriers without FXTAS at visit 1, of which 10 were classified as “*Converters”* because they developed clear FXTAS symptomology at visit 2 (FXTAS stage score was 0–1 at visit 1 and ≥2 at visit 2; mean age at visit 1 = 58.81, *SD* = 6.17 years). We then selected 10 “*Non-converters”* (FXTAS stage score was 0–1 at both visit 1 and visit 2; mean age at visit 1 = 57.95, *SD* = 7.43 years), who were not showing signs of FXTAS, using one-one age matching to use as a comparison group.

**Table 1:** Participant demographics.

	**Controls**	**Premutation without FXTAS**	**Premutation with FXTAS**	**ANOVA *p*-value**
**VISIT 1**				
*n*	61	45	53	
Age (years)	60.75 (9.95)	59.54 (8.17)	66.16 (6.79)	0.001^a^^,^^b^
CGG	29.54 (4.56)	86.11 (24.46)	90.30 (16.41)	0.001^a^^,^^c^
mRNA	1.25 (0.22)	2.49 (0.86)	2.63 (0.62)	0.001^a^^,^^c^
**VISIT 2**				
*n*	21	25	27	
Age (years)	58.59 (9.03)	57.83 (7.96)	68.01 (6.87)	0.001^a^^,^^b^
CGG	29.94 (4.27)	86.72 (23.95)	87.81 (16.56)	0.001^a^^,^^c^
mRNA	1.22 (0.25)	2.47 (0.74)	2.64 (0.66)	0.001^a^^,^^c^

*Numbers in parentheses are standard deviations*.

a *p < 0.05 for comparison between Controls and FXTAS*.

b *p < 0.05 for comparison between Premutation carriers without FXTAS and FXTAS*.

c *p < 0.05 for comparison between Controls and Premutation carriers without FXTAS*.

All participants were fluent English speakers, with no history of any serious medical or neurological conditions, including history of alcoholism or drug abuse.

CGG analysis was completed using genomic DNA isolated from peripheral blood lymphocytes using a combination of PCR and Southern blot analysis as previously described (Tassone et al., 2008; Filipovic-Sadic et al., 2010). Measurements of *FMR1* mRNA expression levels were carried out by qRT-PCR as previously detailed (Tassone et al., 2000).

### MRI acquisition

High resolution structural MRIs were acquired on a 3T Siemens Trio Scanner using a 32-channel head coil and a T1-weighted 3D MPRAGE sequence between May 2010 and June 2017 using the following scan sequence: TR = 2,170 ms, TE = 4.86 ms, flip angle = 7°, FoV = 256 mm^2^, 192 slices, 1 mm slice thickness. The scans were first aligned along the anterior-posterior commissure line using acpcdetect (http://www.nitrc.org/projects/art) (Ardekani and Bachman, 2009). For failed cases, manual alignment was performed using DTI Studio (http://www.mristudio.org) (Mori et al., 1999). Then MRI bias field correction was performed using N4 (http://stnava.github.io/ANTs/) (Tustison et al., 2010).

### MRPI analysis

A series of independent raters (two per measure) who were blinded to the participant age and group, visit quantitatively assessed all MR images for the four MRPI measurements (pons and midbrain cross-sectional areas, as well as MCP and SCP widths) based on methods previously described (Nicoletti et al., 2006; Quattrone et al., 2008) and detailed below.

**Figure 1:**
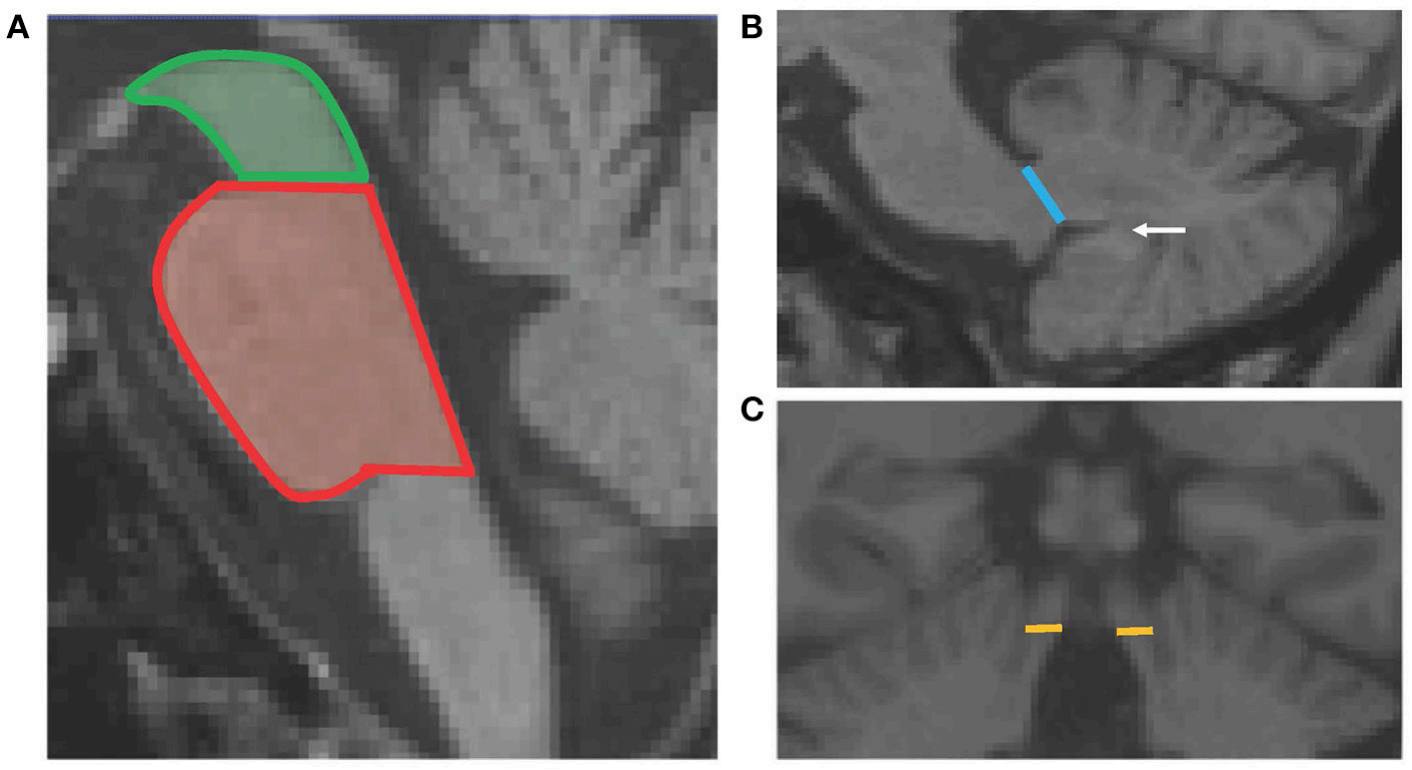
Schematic diagram showing the four MRPI measurements. Panel **(A)** shows the horizontal lines of the superior pontine notch which divides the midbrain (green) and pons (red) cross-sectional areas. Panel **(B)** shows the line drawn to define the MCP width, which is the linear distance between the peripeduncular cerebrospinal fluid spaces of pontocerebellar cisterns. The white arrow points to the white matter connecting the cerebellar tonsil to the deep white matter of the cerebellum. Panel **(C)** shows the horizontal lines which delineate the SCP width, at the coronal slice where the SCP first separates from the inferior colliculi.

The pons and midbrain areas were assessed on the mid-sagittal slice, where horizontal lines were drawn through the superior and inferior pontine notches. The midbrain was measured as the area above the superior pontine line—midbrain tegmentum, while the pons was the area between the two horizontal lines of the superior and inferior notches (Figure 1A).

The width of both left and right MCPs were measured on parasagittal slices. The linear distance of the MCP was delineated by the peripeduncular cerebrospinal fluid spaces of pontocerebellar cisterns, where the pons was still “intact” and the cerebellum was fully formed (white matter connecting the cerebellar tonsil was present; Figure 1B). Finally, the widths of both the left and right SCPs were measured on oblique coronal slices, at the midpoint of the SCP, when it first became separated from the inferior colliculi. The linear distance between the medial and lateral SCP borders was measured (Figure 1C). For both MCP and SCP widths, a mean score was calculated by averaging the left and right measurements for each participant.

The interrater reliability coefficients were excellent for each of the four MRPI scores ranging between 0.986 and 0.993. The mean score of the raters were used for further analysis.

Along with the individual MRPI measurement scores, pons-midbrain area ratio (Pons/Midbrain) and MCP-SCP width ratio (MCP/SCP) were calculated, along with the MRPI [(Pons/Midbrain)^*^(MCP/SCP)] (Quattrone et al., 2008).

### Statistical analysis

Each MRPI measurement and ratio score was assessed for normality for each group (using skewness and kurtosis) and equal variances (Levene test). Age correlated significantly with all MRPI measurements/ratios except for SCP width (Table S1). Therefore, comparisons of MRPI scores were conducted with ANCOVA analysis (age as a covariate, except for SCP width where an ANOVA was used) using Bonferroni corrections for *post-hoc* analyses for group.

To examine the relationships between the MRPI measurements/ratios and both molecular and clinical measures in premutation carriers with and without FXTAS (combined group), scatter plots were used to assess linearity of relationship and were followed by hierarchical regression analysis.

To assess change in MRPI scores between visits across the three groups, a 2 × 3 repeated measures ANCOVA (age as a covariate, except for SCP width where an ANOVA was used) was used. Finally, to assess whether MRPI measurements and ratios may be a useful biomarker for FXTAS, independent *t*-tests and paired samples *t*-tests were used to compare the sub-samples of *Converters* and *Non-converters* at each visit, and between visits for each group.

A significance level of *p* < 0.007 was set for all group and regression analyses, considering a Bonferroni correction for the seven MRPI measurements/ratios examined herein. Bonferroni *post-hoc* analyses were set at *p* < 0.050.

## Results

### Cross-sectional analysis of MRPI measurements and ratios

**Figure 2:**
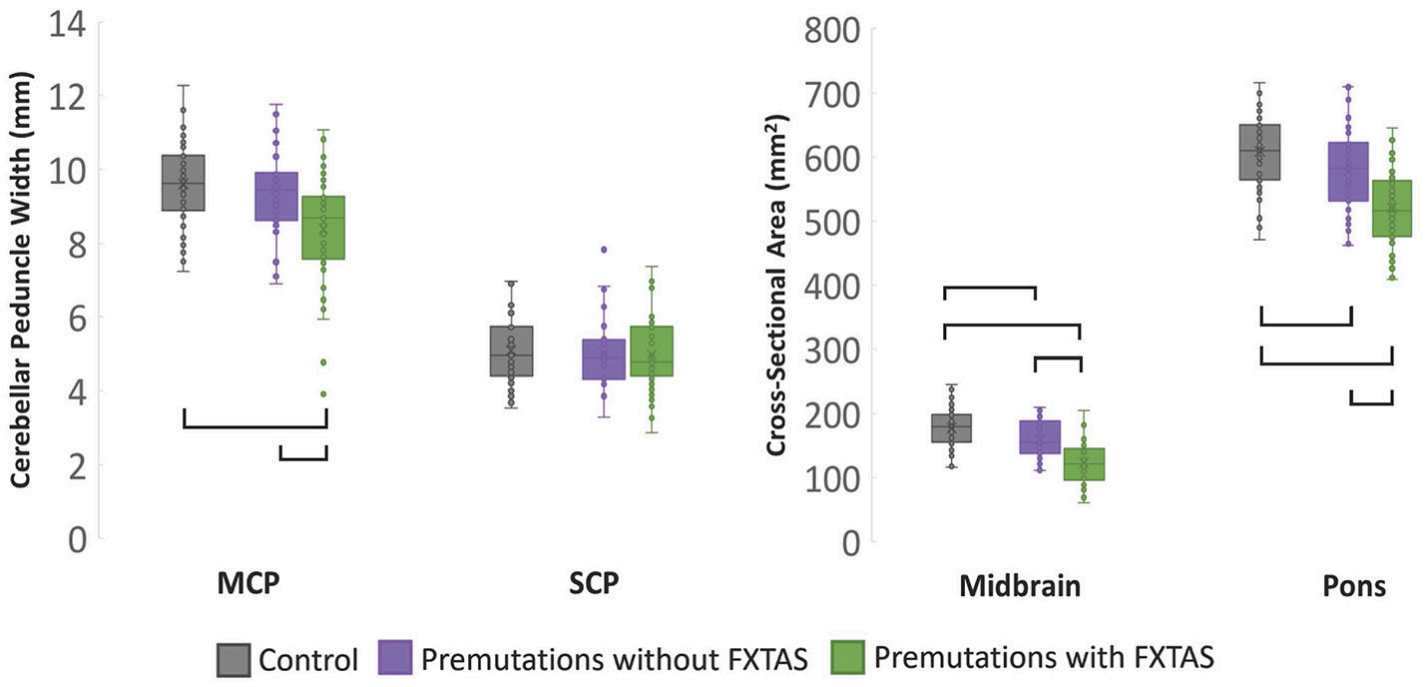
Cross-sectional analysis of MRPI measurements at time 1, with age as a covariate. Brackets represents group differences where *p* < 0.05 using *post-hoc* Bonferroni correction.

MCP width, midbrain and pons cross-sectional area as well as pons/midbrain ratio yielded significant main effects for group (*p* < 0.001) (Table 2). *Post-hoc* pairwise comparisons revealed that patients with FXTAS demonstrated significantly reduced MCP width, midbrain and pons cross-sectional areas and increased pons/midbrain ratio (all *p*s < 0.001) compared to controls (Figure 2). Patients with FXTAS also demonstrated significantly reduced MCP width, midbrain and pons cross-sectional areas (all *p*s < 0.001) and increased pons/midbrain ratio (*p* < 0.002) when compared to premutation carriers without FXTAS (Figure 2). Compared to controls, premutation carriers without FXTAS had reduced midbrain (*p* < 0.001) and pons (*p* < 0.006) cross-section areas (Figure 2).

**Table 2:** Summarized statistics (mean and standard deviations) and ANCOVA/ANOVA statistics for the main effect of group when comparing controls and premutation carriers with and without FXTAS.

	**Controls**	**Premutation Carriers without FXTAS**	**Premutation Carriers with FXTAS**	**F-statistic for group**	***p-*value for group**	***η^2^* for group**
MCP width	9.61 (1.12)	9.42 (1.06)	8.38 (1.41)	16.2	<0.001	0.172^∧^^#^
SCP width	5.09 (0.82)	5.02 (0.94)	4.97 (0.99)	0.25	0.782	0.003
Midbrain	177.4 (29.4)	159.7 (28.6)	122.8 (31.5)	48.3	<0.001	0.382^*^^∧^^#^
Pons	608.5 (56.9)	577.7 (62.4)	519.9 (60.1)	31.9	<0.001	0.290^*^^∧^^#^
SCP/MCP	1.94 (0.40)	1.93 (0.39)	1.72 (0.35)	5.44	0.005	0.065^∧^^#^
Pons/Midbrain	3.51 (0.61)	3.69 (0.55)	4.43 (0.92)	25.6	<0.001	0.248^∧^^#^
MRPI	6.84 (2.01)	7.12 (1.67)	7.60 (1.99)	2.24	0.110	0.028^∧^

*Values in parentheses represent standard deviations. η^2^ represents partial eta squared*;

* *a significant group difference between controls and premutation carriers without FXTAS (p ≤ 0.05 post-hoc)*;

∧ *a significant group difference between controls and premutation carriers with FXTAS (p ≤ 0.05 post-hoc)*;

# *a significant group difference between premutation carriers with and without FXTAS (p ≤ 0.05 post-hoc)*.

Interestingly, a main effect for age was found for midbrain cross-sectional area and pons/midbrain ratio (*p* < 0.007) following correction for multiple comparisons, as well as for pons cross-sectional area prior to correction (*p* = 0.043). In these instances, when an age × group interaction was added to the model, the main effect of group disappeared, with the exception of pons/midbrain ratio where it remained significant prior to correction for multiple comparisons (*p* = 0.008), where the FXTAS group had a higher ratio than both the control group (*p* < 0.001) and premutation carriers without FXTAS group (*p* = 0.015). Premutation carriers without FXTAS showed a trend toward increased pons/midbrain ratio compared to controls (*p* = 0.069). However, a significant age × group interaction was found for the pons/midbrain ratio (*p* = 0.001), whereby the steepest rate of incline was for the FXTAS, followed by the premutation without FXTAS group. Age remained a significant predictor of midbrain cross-sectional area (*p* < 0.001) and pons/midbrain ratio (*p* < 0.001) following correction, as well as pons cross-sectional areas (*p* = 0.012) prior to correction for multiple comparisons.

#### Relationships between MRPI measurements/ratios, FXTAS stage, and molecular markers for premutation carriers

The MRPI measurements and ratios for all premutation carriers combined revealed significant relationships with the FXTAS stage scores (Table 3), but not for CGG or *FMR1* mRNA (*p* > 0.007) at visit 1. However, prior to correcting for multiple comparisons, increased CGG repeat length was found to be associated with reduced midbrain cross-sectional area (β = −0.192, *SE* = 0.075, *p* = 0.012), and increased pons/midbrain ratio (β = 0.210, SE = 0.800, *p* = 0.010), as well as increased *FMR1* mRNA and increased pons/midbrain ratio (β = 0.172, *SE* = 0.084, *p* = 0.042).

**Table 3:** Relationships between MRPI measurements/ratios and the FXTAS stage scale for all premutation carriers with and without FXTAS.

	**β**	**SE**	***p-*value**
MCP width	−0.513	0.098	<0.001
SCP width	−0.231	0.116	0.050
Midbrain cross-sectional area	−0.407	0.077	<0.001
Pons cross-sectional area	−0.472	0.099	<0.001
MCP/SCP	−0.185	0.108	0.091
Pons/Midbrain	0.324	0.087	<0.001
MRPI	0.089	0.115	0.441

*A hierarchical regression model was used, with age included for all analyses except SCP width. β = standardized regression coefficient*.

#### Comparison of MRPI measurements/ratios between converters and non-converters

**Figure 3:**
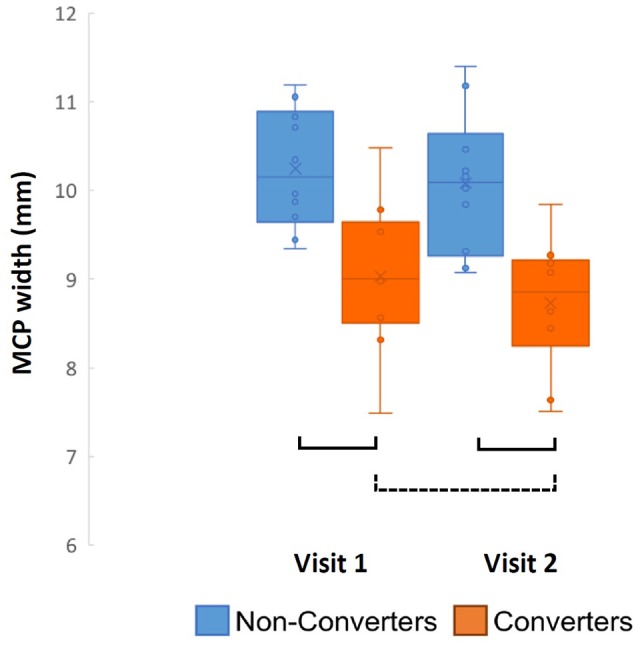
Converter and non-converter group comparison of MCP width over time. Solid lines represent *p* < 0.007 and surviving Bonferroni correction for multiple comparisons, while the dotted line represents *p* < 0.05 and not surviving Bonferroni correction.

When comparing the Converters and Non-converters MRPI scores, converters had significantly decreased mean MCP width at both visits (Time 1: *p* = 0.002, Cohen's *d* = 1.587; Time 2: *p* < 0.001, Cohen's *d* = 1.762), in which there was almost no overlap in scores between groups at each visit (Figure 3). Pons cross-sectional area was also reduced in Converters compared to Non-converters at both visits (Time 1: *p* = 0.013, Cohen's *d* = 1.124; Time 2: *p* = 0.010, Cohen's *d* = 1.279), although these differences did not survive Bonferroni correction for multiple comparisons (Table 4).

**Table 4:** Demographics, summarized statistics (mean and standard deviations) and comparisons (*p* value) between premutation carrier *converters* and *non-converters*.

	**Visit 1**			**Visit 2**		
	**Converters**	**Non-Converters**	***p***	**Converters Mean**	**Non-Converters**	***p***
	**Mean (*SD*)**	**Mean (*SD*)**		**Mean (*SD*)**	**Mean (*SD*)**	
MCP	9.03 (0.85)	10.2 (0.67)	0.002	8.73* (0.74)	10.1 (0.80)	0.001
SCP	4.94 (0.55)	5.31 (1.30)	0.412	5.08 (0.70)	5.18 (1.06)	0.812
Midbrain	153.9 (25.9)	173.4 (30.9)	0.146	150.7 (23.0)	170.3 (32.0)	0.132
Pons	549.6 (65.4)	616.4 (39.7)	0.013	544.6 (68.8)	618.4 (43.6)	0.010
MCP/SCP	1.86 (0.32)	2.02 (0.47)	0.368	1.72 (0.36)	2.00 (0.32)	0.080
Pons/Midbrain	3.62 (0.44)	3.65 (0.59)	0.911	3.64 (0.41)	3.73 (0.61)	0.706
MRPI	6.74 (1.58)	7.33 (1.94)	0.470	6.29 (1.70)	7.52 (1.96)	0.151

Values in parentheses represent standard deviations. *Indicates that a paired samples t-test between visits is p = 0.032, and significant prior to correction for multiple comparisons.

### Longitudinal analysis of MRPI measurements and ratios

MRPI scores were not found to change significantly between controls, premutation carriers without FXTAS and premutation carriers with FXTAS between visits 1 and 2 except for midbrain cross-sectional area (Main effect for group: *F* = 3.45, *p* = 0.037). Premutation carriers without FXTAS showed a greater change in midbrain cross-sectional area between visit 1 and visit 2 than premutation carriers with FXTAS (*F* = 6.76; *p* = 0.011).

#### Changes in MRPI measurements in FXTAS converters

To examine whether MRPI scores are useful biomarkers for newly developing FXTAS cases, a mixed-effects regression model was used to examine change in MRPI scores between visits for those premutation carriers who received a rating of 0 or 1 (no or equivocal tremor/ataxia) on the FXTAS stage scale at visit 1, but received a 2 or more on the same scale (clear tremor/ataxia with some interference in functioning) at their second visit (*Converters, n* = 10). No significant differences in MRPI measurements/ratios between visit 1 and visit 2 were perceptible (*p* > 0.05).

Following these analyses, a series of paired samples *t*-test were used to assess change in MRPI measurement/ratios between visits for *Converters* and *Non-converters*, separately for each variable. MCP width was the only MRPI measurement/ratio to change significantly between visits for the Converters (*p* = 0.032, Cohen's *d* = 0.835), although this change did not survive Bonferroni correction for multiple comparisons (Table 4; Figure 3). No significant changes were found for the age-matched Non-converters (*p* > 0.007).

## Discussion

FXTAS is a progressive and severe neurodegenerative disease, with no established prodromal or risk biomarkers. This study shows that specific and reliably-measured neuroanatomical changes may precede and possibly predict clinical symptom development. First, decreasing midbrain and pons cross-sectional areas may be an *FMR1* premutation phenotype associated with accelerated neurodegeneration. Further, and perhaps more interestingly, decreasing MCP width appears to be sensitive to early structural changes associated with FXTAS development. With larger validation and sensitivity studies, this measure could represent a clinically useful biomarker for FXTAS risk.

The hummingbird signal (increase in pons to midbrain cross-sectional area ratio), suggests advanced atrophy of the midbrain compared to the pons (Kato et al., 2003). In this study, we found systematic atrophy of both the midbrain and pons, and thus an increase pons/midbrain ratio in both *FMR1* premutation carriers with and without FXTAS, as well as accelerated age-related increase in the pons/midbrain ratio in the FXTAS group (and a trend for the *FMR1* premutation carriers without FXTAS group) compared to controls. However, the close to significant relationships between increased CGG repeat length, decreased midbrain cross-sectional area, and increased pons/midbrain ratio, may suggest that the hummingbird signal and preferential atrophy of the midbrain is more likely to occur in *FMR1* premutation carriers with high CGG repeats, who have previously been found to have the greatest FXTAS-related movement impairment (Leehey et al., 2008).

*FMR1* premutation age-related atrophy has been previously detected in patients with FXTAS compared to controls and premutation carriers without FXTAS when examining brainstem volumes (combined volume of midbrain, pons, and medulla) (Wang et al., 2017). Thus, the findings of the current study compliment these prior findings, and extend them to suggest that FXTAS related neurodegeneration may accelerate otherwise normal age-related decreases in midbrain and pons cross-sectional areas, with the hummingbird signal (or increased midbrain compared to pons atrophy) appearing in those with greater CGG repeat lengths.

In addition, the pons is an important relay area for neural pathways with cortical—cerebellar links. While changes between visits in pons cross-sectional area were not found in this study, the tendency for reduced volumes (even if they did not survive multiple correction) between *Converters* and *Non-converters* is striking. Indeed, pontine linear dimensions and white matter density reductions have been previously documented in older *FMR1* premutation carriers with FXTAS (Brunberg et al., 2002; Moore et al., 2004). Hence, with a larger sample of *converters* and greater statistical power, measuring the cross-sectional area of the pons may be fruitful for FXTAS diagnosis and risk assessment.

The MCP has been recognized as a critical area of pathology and a key MRI indicator of FXTAS (Jacquemont et al., 2003; Rivera et al., 2010; Hall et al., 2014). However, the neuropathology that causes the increased T2 MRI signal—or hyperintensity—is currently unknown and does not exclusively occur in FXTAS. Indeed, the MCP pathway itself plays an important role in cortical connections to the cerebellum, and thus it is logical that the MCP width significantly correlated with FXTAS stage score, which emphasizes tremor and ataxia. Although speculative, perhaps the lack of CGG or *FMR1* mRNA associations with MCP width means that the microstructure changes that cause the MCP signal may be in part due to decreased tract integrity resulting from *FMR1* exon 1 methylation mediated pathways as previously hypothesized (Shelton et al., 2017), rather than those directly related to *FMR1* expression levels. We are currently examining the relationships between white matter hyperintensities, microstructural integrity and morphological changes in the MCP. It is critical to better understand the neuropathological changes that contribute to the development and progression of FXTAS symptoms.

Out of the four measurements and three ratios, the MCP width appears to be the best biomarker for FXTAS conversion in those *FMR1* premutation carriers most at risk, which survived a strict Bonferroni correction. This stems from both the cross-sectional analysis of the three groups at visit one, and comparison of *Converters* and *Non-converters*. Firstly, it was the only measure in which group differences were revealed between patients with FXTAS and both controls and premutation carriers without FXTAS, yet no difference was found between controls and premutation carriers without FXTAS. This is unlike the results from the midbrain, pons areas and pons/midbrain ratio, where differences were found between controls and premutation carriers without FXTAS. Further, MCP width was the only measure that reliably detected a difference between converters and non-converters, with an indication that it even decreased as FXTAS symptoms develop over an average period of 2.16 years. Together, these results suggest that MCP width may be a clinically useful biomarker for FXTAS, as it was sensitive to FXTAS onset and progression, but not the *FMR1* phenotype more generally. Further, the findings highlight the importance of continually tracking the MCP width of *FMR1* premutation carriers to verify its predictive value for FXTAS development.

While the results reported here are compelling, the study is not without limitations. Given the longitudinal aspect of this project, it is critically important that we continue to follow and track the progression of these biomarkers in conjunction with various neurological and neuropsychological symptoms, in particular motor control, balance and ataxia, and executive function (Shickman et al., 2018). Indeed, it will be critical to confirm whether or not the current group of non-converters remains asymptomatic, and if these findings remain in a larger cohort of Converters and Non-converters. Thus as we continue to collect follow-up scans, we expect to see more *FMR1* premutation carriers convert to FXTAS, which will increase the power to detect change over time, and perhaps predict how soon conversion may occur.

Overall, these results support the hypothesis that specific neuroanatomical changes precede and are related to FXTAS symptom severity. Indeed, it appears that atrophy of the midbrain and pons cross-sectional area may be a common phenotypic characteristic for *FMR1* premutation carriers, while reductions in MCP width may be prognostic to FXTAS symptoms and progression. While we endeavor to assess to clinical viability of MCP width as a biomarker of FXTAS through our ongoing research, we compel clinicians to monitor and assess changes in the MCP, primarily for atrophy in width and hyperintensities, as well as other FXTAS radiological signs in premutation carriers at risk for FXTAS or with subtle non-specific clinical changes e.g., (Shickman et al., 2018). In sum, MCP width in *FMR1* premutation carriers may be a candidate biomarker to clinically identify patients in prodromal and early stages of FXTAS to help guide candidates for treatment and perhaps monitor response.

## Author contributions

AS developed the methodology for and supervised the brain measurements and wrote the first draft of the manuscript. JW contributed conceptually to the data analysis strategy, and wrote sections of and edited the manuscript. EF performed a significant portion of the brain measurements, contributed to the analysis strategy, and edited the manuscript. FT contributed conceptually to the study design, provided molecular measurements for the data analysis and edited the manuscript. AC and LF performed significant portions of the brain measurements and edited the manuscript. RH contributed conceptually to the study design and provided the primary clinical measurements of FXTAS. EF helped develop the data analysis strategy, performed some of the data analysis, and wrote sections of and edited the manuscript. DH contributed to the design of the study, supervised the collection of clinical assessment data, and wrote sections of and edited the manuscript. SR contributed to the design of the study, supervised neuroimaging data collection and analysis, and wrote sections of and edited the manuscript.

### Conflict of interest statement

RH has received funding from Zynerba, Fulcrum and Ovid for consultation regarding treatment of fragile X syndrome and from Neuren and Marinus for carrying out fragile X treatment studies. DH has received funding from Ovid, Fulcrum, and Autifony for consultation regarding fragile X treatment studies. The remaining authors declare that the research was conducted in the absence of any commercial or financial relationships that could be construed as a potential conflict of interest.

## Abbreviations

FXTAS: Fragile X-associated tremor/ataxia syndrome
MCP: Middle Cerebellar Peduncle
MRPI: Magnetic Resonance Parkinsons Index.
