# Supplementary material for: Middle Cerebellar Peduncle Width—A Novel MRI Biomarker for FXTAS?
Source: Front Neurosci. 2018 Jun 25;12:379. doi: 10.3389/fnins.2018.00379 (PMC6026659; doi:10.3389/fnins.2018.00379)
Supplement: Supplementary file 1 [file Data_Sheet_1.PDF]

Supplementary material

Table S1: Correlation between age and MRPI measurements/ratios for all participants at time 1.

		MCP width	SCP width	Midbrain area	Pons area	MCP/SCP	Pons/Midbrain	MRPI
**Age**	*r*	-.196	.066	-.585	-.275	-.199	.569	.282
	p	.013	.410	.000	.000	.012	.000	.000

Note: for all analyses *n*=158.
